# Unveiling the role of GAS41 in cancer progression

**DOI:** 10.1186/s12935-023-03098-z

**Published:** 2023-10-18

**Authors:** Kangkang Ji, Li Li, Hui Liu, Yucheng Shen, Jian Jiang, Minglei Zhang, Hongwei Teng, Xun Yan, Yanhua Zhang, Yong Cai, Hai Zhou

**Affiliations:** Department of Central Laboratory, Binhai County People’s Hospital, Yancheng, 224000 China

**Keywords:** GAS41, Cancer, Reader, Histone modifications, Inhibitor

## Abstract

GAS41, a member of the human YEATS domain family, plays a pivotal role in human cancer development. It serves as a highly promising epigenetic reader, facilitating precise regulation of cell growth and development by recognizing essential histone modifications, including histone acetylation, benzoylation, succinylation, and crotonylation. Functional readouts of these histone modifications often coincide with cancer progression. In addition, *GAS41* functions as a novel oncogene, participating in numerous signaling pathways. Here, we summarize the epigenetic functions of GAS41 and its role in the carcinoma progression. Moving forward, elucidating the downstream target oncogenes regulated by GAS41 and the developing small molecule inhibitors based on the distinctive YEATS recognition properties will be pivotal in advancing this research field.

**Supplementary Information:**

The online version contains supplementary material available at 10.1186/s12935-023-03098-z.

## Introduction

**Fig. 1:**
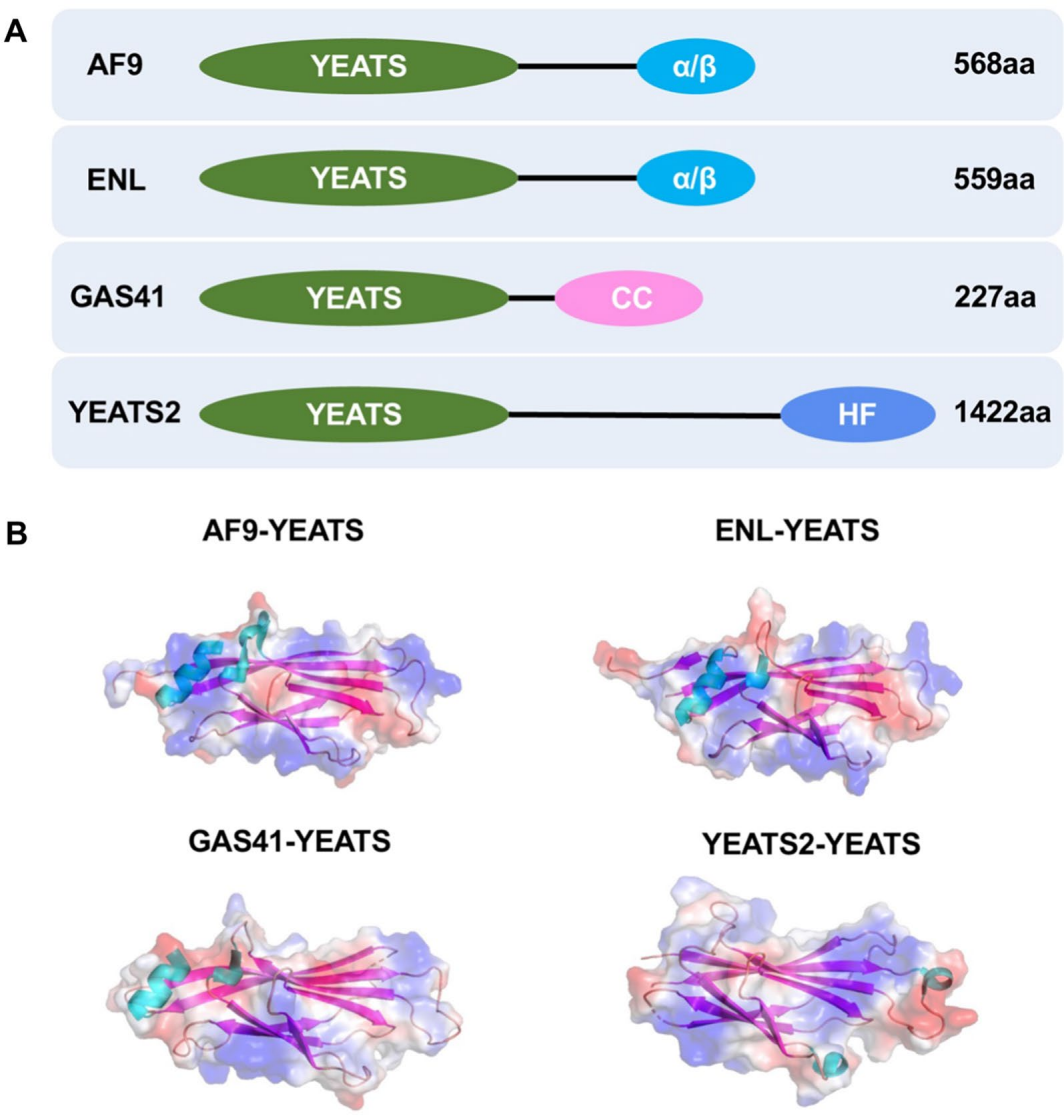
Structure of the human YEATS family. **A** Schematic diagram of the one-dimensional structure of the YEATS family. GAS41 is the shortest one, comprising 227 amino acids and a CC region at the C-terminus, whereas YEATS2 is the longest amino acid sequence and contains a histone fold at the end. **B** PDB structure of the YEATS domain. File from PBD: 7eic, 5j9s, 7eif, 7eie. ‘aa’: amino acid, ‘CC’: coiled-coil, ‘α/β’: α helix and β hairpin motif, 'HF': histone fold

**Fig. 2:**
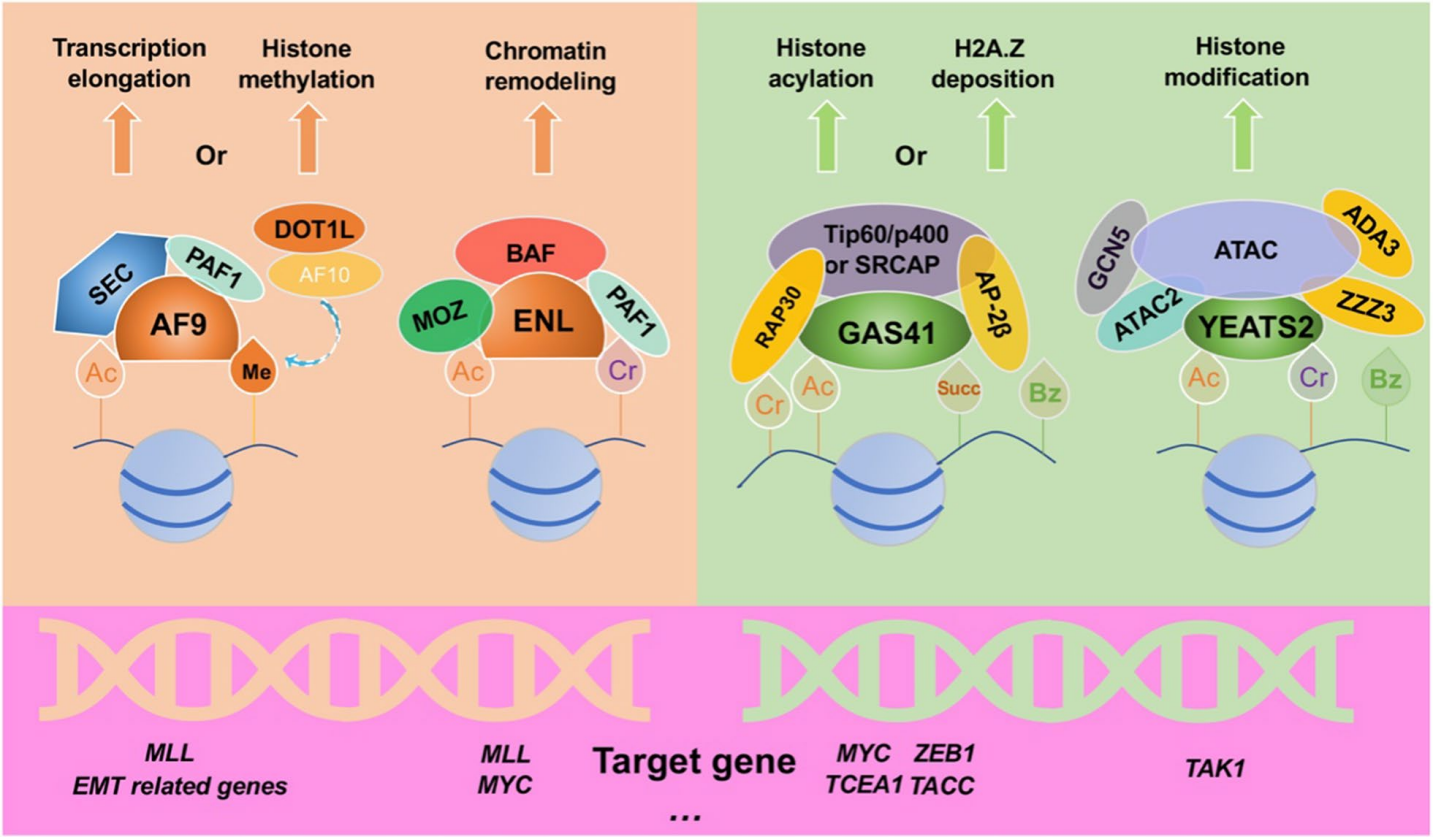
Epigenetic functions of the YEATS family Map of biological functions and mechanisms of the YEATS family. YEATS acts as an epigenetic reader to recognize histone modifications. *Ac* acetylation, *Me* methylation, *Cr* crotonylation, *Bz* benzoylation, *Succ* succinylation

The human YEATS family comprises four members: all1-fused genes from chromosome 9 (AF9), eleven-nineteen-leukemia (ENL), glioma amplified sequence 41 (GAS41), and YEATS2 (Fig. 1). The acronym "YEATS" stems from the initial letters of five related domain proteins (Yaf9, ENL, AF9, Taf14, and Sas5) [1]. The family exhibits a conserved YEATS domain at the N-terminus (Fig. 1) and plays diverse roles in chromatin dynamics, histone modifications, and gene regulation [2]. YEATS primarily governs transcriptional elongation, histone modification, histone variant (H2A.Z) deposition, and chromatin remodeling in epigenetics (Fig. 2). AF9 and ENL act as fusion chaperones for human mixed lineage leukemia proteins (MLL) resulting from chromosomal translocations and contribute to acute myeloid leukemia [3, 4]. At the molecular level, AF9 recruits transcription factors such as the super elongation complex (SEC) [5] and polymerase-associated factor 1 (PAF1) [6] to recognize acetylation modifications and regulate downstream transcriptional elongation (Fig. 2). Acetylation is one of diverse acylation. The type of acylation depends primarily on the acyl groups attached to the lysine residue, including acetyl-, succinyl-, malonyl-, crotonyl-, β-hydroxybutyryl-, lactyl-, myristoyl-, and palmitoyl-CoA [7]. Furthermore, AF9 is involved in histone methylation through AF10 [8] and histone-lysine N-methyltransferase DOT1L [9] (Fig. 2). ENL, in combination with the monocytic leukemia zinc (MOZ) complex [10], and the BRG1-associated factor (BAF) [11] facilitates histone acetylation reading and chromatin remodeling (Fig. 2). Extensive research has unveiled *MLL* as a classical downstream target gene of AF9 and ENL [12, 13]. Additionally, AF9 targets a crucial set of genes associated with epithelial-to-mesenchymal transition (EMT) [14]. Apart from MLL, the well-known proto-oncogene MYC is also targeted by ENL [14], and it holds great potential as a candidate for cancer therapy [15]. YEATS2 acts as a distinct reader of histone crotonylation [16] and serves as a novel oncogene in various cancers [17–19]. YEATS2 has also been found to activate the TAK1/NF-κB and PI3K/AKT signaling pathways, influencing cancer cell survival [20, 21]. To delve into the underlying mechanisms, YEATS2 is known to recruit the Ada-two-A-containing (ATAC) complex [22] to identify specific histone modifications and facilitate histone modifications (Fig. 2). It is noteworthy that ATAC functions as a transcriptionally active complex involved in chromosome remodeling [23], comprising transcription factors such as general control non-depressible protein 5 (GCN5) [24], ATAC2, alternation/deficiency in activation-3 (ADA3) [25] and zinc finger ZZ-type containing 3 (ZZZ3) [26]. YEATS2 is highly expressed in human pancreatic ductal adenocarcinoma (PDAC) and can positively regulate the growth, survival, and tumorigenesis of PDAC cells [21]. The binding of YEATS2 is crucial for maintaining transforming growth factor beta-activated kinase 1 (TAK1) activation and NF-κB transcriptional activity. Of importance, YEATS2 promotes NF-κB transcriptional activity through modulating TAK1 abundance and directly interacting with NF-κB as a co-transcriptional factor [21]. GAS41, a polymorphic protein, plays a crucial role in recognizing lysine-acylated histones through its YEATS domain [27]. GAS41 selectively recognizes histone modifications that are frequently associated with downstream diseases, including cancer [28]. Complex structural studies have revealed that the YEATS domain utilizes a common binding pocket to interpret distinct lysine acylation modifications [29]. The acylated lysine side chain extends into the YEATS domain, where it interacts with a set of aromatic residues, forming the well-known aromatic sandwich model (also referred to as ‘π-π-π’) [28]. Notably, the aromatic amino acids W93, F96, and Y74 play a central role in the representative structure of the GAS41-YEATS-H3K27ac complex [30, 31]. Intriguingly, GAS41 has been characterized as a novel oncogene with indications of aberrant amplification in various cancers [32]. Importantly, GAS41 has been found to target downstream regulators such as Zinc finger E-box-binding homeobox 1 (ZEB1) [33], transforming acidic coiled-coil 1 (TACC) [34], and (Transcription elongation factor A protein 1) TCEA1 [35] to modulate cancer progression (Fig. 2). In conclusion, GAS41 exhibits dual identities in the organism, functioning either as a transcription factor involved in epigenetic regulation or as a signal transduction protein participating in intracellular signal transduction within cancer cells.

### The oncogenic potential of GAS41

*GAS41*, originating from the chromosome 12q13-15 region of glioma cells, is frequently amplified in gliomas [36]. Notably, most gene amplifications in gliomas occur in advanced tumor stages. However, the early-stage amplification of *GAS41* suggests its significant oncogenic activity during early tumor progression [37]. Overexpression of GAS41 has been observed in various cancer types, such as breast cancer [38], non-small cell lung cancer [31, 39], hepatocellular carcinoma [35, 40], pancreatic cancer [41, 42], gastric cancer [43, 44], colorectal cancer [45, 46], atypical adipose carcinoma [47], ovarian cancer [48], and uterine fibroids [49, 50]. These findings have prompted researchers to reevaluate the oncogenic properties of *GAS41*.

### GAS41 as a transcription factor

Initially identified in the nucleus, GAS41 demonstrates transcription factor properties [51]. Notably, GAS41 shares significant sequence homology with the human mixed spectrum leukemia translocation protein (MLLT1/MLLT3), commonly referred to as ENL and AF9 [13, 52]. Studies have revealed that GAS41 participates in the assembly of two multi-subunit complexes, namely tat-interactive protein 60 / e1a-binding protein p400 / transcription/transformation domain-associated protein (TIP60/p400/TRRAP) and SNF2-related CBP activator protein (SRCAP) [53]. The involvement of activating enhancer-binding protein 2-beta (AP-2β) [54] and RNA polymerase-associated proteins 30(RAP30) [55] is essential for the recognition of histone acylation modifications by GAS41 (Fig. 2). By recognizing histone modifications, GAS41 facilitates the participation of histones in nucleosome remodeling, thereby influencing gene transcription [1, 28, 56].

### Role of GAS41 in the context of epigenetic signaling

Epigenetic signaling is the biological process that leads to changes in epigenetic marks such as DNA methylation, histone modifications (Kac, Kcr, Klac, etc.), non-coding RNAs (miRNAs and siRNAs), and chromatin accessibility [57–60]. Functional readouts of histone modifications serve as an important mechanism for epigenetic signaling. GAS41 is involved in epigenetic signaling primarily as a reader recognizing histone modifications [61], including histone acetylation (H3K27ac and H3K14ac) [30, 31], benzoylation (H3K27bz) [62], crotonylation (H3K27cr) [63], and succinylation (H3K122suc) [64]. Histone modifications typically affect nucleosome stability and serve as anchors for chromatin-associated protein complexes [28]. GAS41 likely establishes a signaling axis that links histone modification readouts to H2A.Z deposition. For example, the readouts of H3K27ac and H3K14ac by GAS41 could recruit Tip60/p400 or SRCAP complexes to deposit H2A.Z into specific chromatin regions [30, 31]. GAS41 could recruit the Dot1l-RNA polymerase (Pol) II complex to the gene promoter by recognizing H3K27ac, thereby initiating gene transcription [65]. By binding to H3K27cr, GAS41 can be recruited by MYC to the SIN3A-HDAC1 co-repressor to repress the transcription of p21-related genes [63]. H3K122suc is recognized by GAS41 in a pH-dependent manner and is co-enriched with GAS41 at the p21 promoter [64]. In parallel to histone modifications, GAS41 can also participate in epigenetic signaling through non-coding RNAs. lncAKHE, a long non-coding RNA highly expressed in hepatocellular carcinoma, was found to cooperate with GAS41 to enhance the expression of NOTCH2-related genes [40]. Additionally, GAS41 is a pivotal component of RNAi and has been identified as a potential epigenetic regulator of miR-203, miR-218, and miR-10b [46, 66, 67]. A recent study indicated that HDAC3 mediates transcriptional repression through GAS41 and the co-repressor DMAP1 [68]. Such evidence provides additional context for the epigenetic regulatory functions of GAS41 beyond the well-described mechanisms primarily associated with histone modification.

## GAS41: Advancing cancer research

**Fig. 3:**
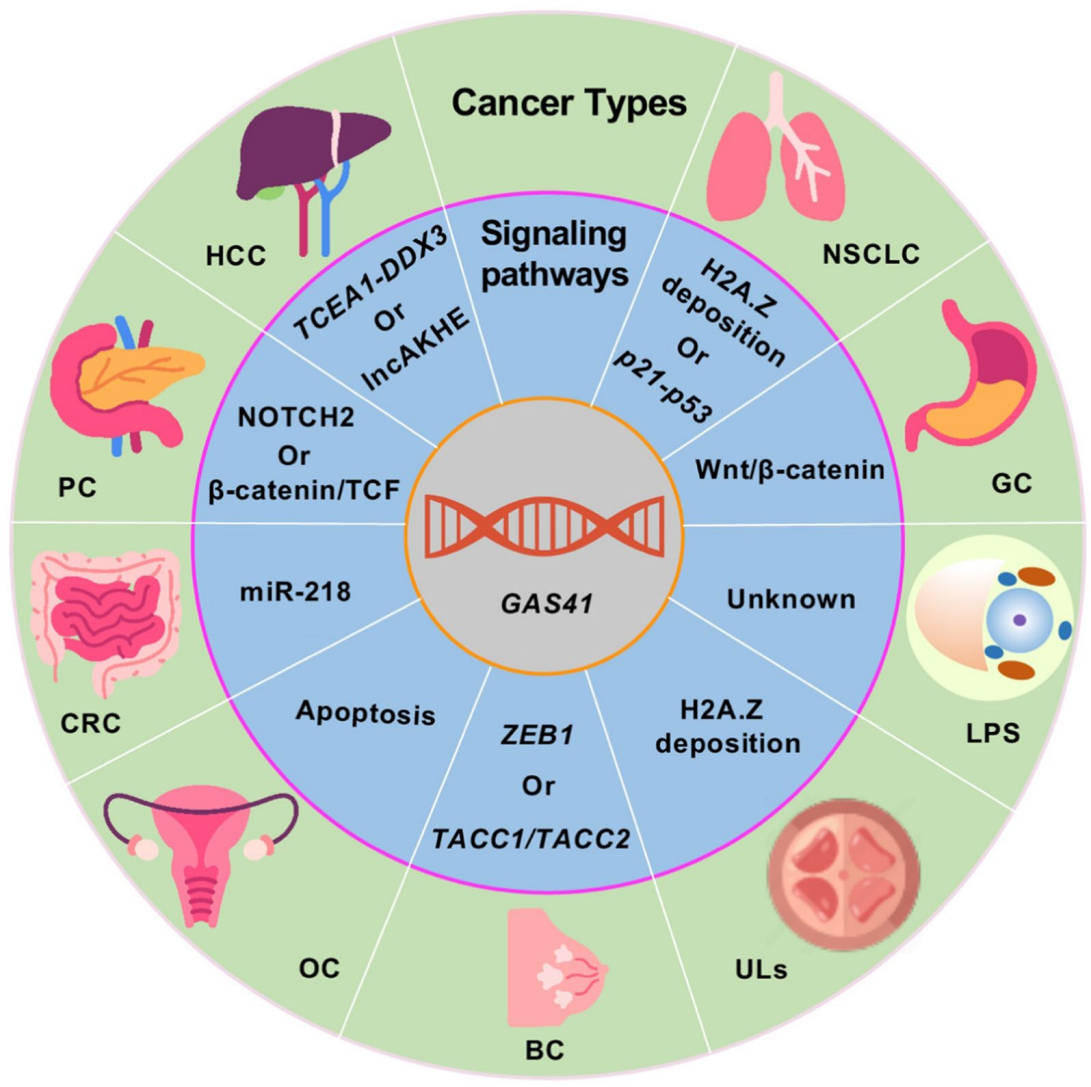
An overview of cancer-associated roles of *GAS41*. An overview of the molecular mechanisms of GAS41 in numerous cancers. *HCC* hepatocellular carcinoma, *BC* breast cancer, *NSCLC* non-small cell lung cancer, *PC* pancreatic cancer, *GC* gastric cancer, *CRC* colorectal cancer, *ULs* uterine fibroids, *LPS* liposarcoma, *OC* ovarian cancer

The exploration of the role of GAS41 in cancer has been a gradual process, with significant attention being directed to this field only in recent years. Initially, Park et al. identified that loss of *GAS41* function resulted in the upregulation of two tumor suppressors, *p14ARF* and *p53* [53]. Building upon these findings, Llanos et al. proposed GAS41 as a robust negative regulator of p53, independent of the chromatin removal or modification complexes [69]. Subsequent investigations revealed a novel oncogenic mechanism of p53 dephosphorylation by GAS41 through the phosphatase specificity of the GAS41-PP2Cβ complex, specifically targeting phosphorylated serine at position 366 of p53 [37]. Studies have shown that increased GAS41 inhibits apoptosis. The collaboration between GAS41 and *lncAKHE* activates the NOTCH2 pathway, which plays a critical role in controlling the apoptosis of hepatocellular carcinoma cells [40]. The coexistence of GAS41 and lncRNA implies a complex regulatory mechanism for GAS41. Fu et.al uncovered that miR-218 sensitized HCT-116/L-OHP (Oxaliplatin) cells to L-OHP-induced cell apoptosis via inhibition of cytoprotective autophagy by targeting GAS41 expression. Xian et.al indicated that the upregulation of GAS41 promotes DNA damage repair and prevents cell death, whereas its downregulation inhibits DNA replication and induces apoptosis [70]. Moreover, GAS41 enhances the proliferation of gastric cancer cells by upregulating its expression to activate the Wnt/β-catenin signaling pathway [43]. However, while many studies have focused on the role of GAS41 in promoting cancer cells, there is still a pressing need to understand its contributions to cancer invasion and metastasis. In this context, we present the existing reported pathways involving GAS41 in cancer to lay the foundation for further investigations on GAS41 (Fig. 3 and Additional file 1: Fig. S1).

### GAS41 in Hepatocellular Carcinoma (HCC)

Hepatocellular carcinoma (HCC) is a highly lethal liver malignancy with a rising global incidence [71]. The challenging aspects of early diagnosis and the aggressive, metastatic, and recurrent nature of HCC contribute to its poor prognosis [72]. Therefore, investigating the key pathways involved in HCC development is crucial, as it may provide valuable insights into identifying early biomarkers and potential therapeutic targets. Previous studies show that TCEA1 is significantly upregulated in HCC [73]. DDX3 was first known for its role in the proliferation and transformation of eternalized human breast cancer epithelial cells [74]. Studies have hinted at DEAD box protein 3 (DDX3) growth-regulatory functions in hepato-carcinogenesis and progression [75, 76]. Upregulation of TCEA1 increased the stability of DDX3 protein and enhanced the proliferation and colony formation of HCC cells [35]. You et al. uncover that GAS41 is significantly upregulated in HCC and correlates with poor prognosis, tumor size, differentiation, and metastasis [35]. GAS41 enhances the transcription of TCEA1 by binding to the TCEA1 promoter, resulting in the upregulation of TCEA1 expression, which stabilizes DDX3 protein and promotes proliferation and colony formation of HCC cells [35]. Furthermore, recent findings highlight the strong expression of lncAKHE in HCC tissues, and its interaction with GAS41 activates the NOTCH2 pathway [40]. These observations strongly support the potential role of GAS41 in HCC (Fig. 3). Consequently, GAS41 emerges as a promising therapeutic target and prognostic indicator for HCC.

### GAS41 in breast cancer (BC)

Breast cancer (BC) is a prevalent malignancy affecting the epithelial cells of the breast, and it ranks as the second most common cancer in women, leading to significant morbidity and mortality [77]. Although BC is often curable in the early stages, its metastatic nature poses significant challenges for treatment. Therefore, there is an urgent need to identify biomarkers associated with metastatic BC [78]. Previous research has established a strong correlation between GAS41 and the breast cancer suppressor TACC [79], with GAS41 shown to interact with TACC1 and TACC2 of the TACC family [80]. However, the exact role of this interaction in the oncogenic process is currently unknown. TACC has been instrumental in BC cell proliferation, and the immunohistochemical status of TACC2 has emerged as a potential prognostic marker of poor prognosis of BC patients [81]. TACC proteins have recently emerged as important players in the complex process of regulating microtubule dynamics during cell division [82, 83]. TACC proteins are usually localized to centrosomes [84] and all phenotypes of altered TACC expression are associated with defects in microtubule stability [85, 86]. Therefore, it is reasonable to speculate that the binding of GAS41 and TACC may affect the cytokinesis process in cancer cells. Notably, overexpression of *GAS41* in BC reinforces the malignant features, especially inducing epithelial-mesenchymal transition (EMT), which contributes to an aggressive phenotype in both *vitro* and *vivo* models [38]. In contrast, the knockdown of *GAS41* suppresses cell growth, promotes mesenchymal-epithelial transformation (MET), and inhibits BC metastasis [38]. The positive regulatory impact of GAS41 on ZEB1 transcription through the recognition of histone H3K27 acetylation (H3K27ac) underlies these biological behaviors [38]. In summary, GAS41 can influence breast cancer progression either through its interaction with the TACC pathway or by regulating *ZEB1* expression (Fig. 3). The documented significance of *GAS41* in BC progression and metastasis underscores the potential therapeutic value of targeting GAS41 expression in BC treatment.

### GAS41 in non-small cell lung cancer (NSCLC)

Non-small cell lung cancer (NSCLC) comprises approximately 80–85% of all lung cancers [87]. Unfortunately, only a small fraction of NSCLC patients are diagnosed at an early stage (stage I/II) when surgical resection is a viable treatment option. The majority of lung cancer patients (more than 60%) present with locally advanced or metastatic disease (stage III/IV) at the time of diagnosis [88]. Pikor et al., through their gene expression analysis, identified GAS41 as a novel candidate oncogene for NSCLC. Moreover, they revealed that GAS41 is an important negative regulator of the *p21-p53* pathway [39]. Frequent amplification of GAS41 in NSCLC has been observed, and its presence is crucial for the survival and transformation of NSCLC cells [31]. Intriguingly, ChIP-seq results have shown that GAS41 co-localizes with H3K27ac and H3K14ac at the promoters of actively transcribed genes. The knockdown of *GAS41* or disruption of the interaction between the YEATS domain and acetylated histones impairs the association of the histone variant H2A.Z with chromatin, thereby inhibiting the growth and survival of NSCLC cells both in *vitro* and in *vivo* [31]. These findings suggest that GAS41 influences the deposition of H2A.Z on chromatin by recognizing histone acetylation modifications, ultimately regulating the promotion of NSCLC (Fig. 3). Recent studies have reported the development of a new dimeric analog with a nanomolar activity that targets lung cancer cells by blocking the interaction between GAS41 and acetylated histone H3. This analog effectively inhibits the growth of NSCLC cells [27]. The investigation of the regulatory mechanisms of *GAS41* in NSCLC and the identification of small molecule inhibitors could offer a promising framework for the treatment of NSCLC.

### GAS41 in pancreatic cancer (PC)

Pancreatic cancer (PC) is a highly aggressive malignancy with a discouraging 5-year overall survival rate of only 11% [89]. In the early stages, PC often presents with no noticeable symptoms, and clinical manifestations typically appear once the tumor invades surrounding tissues or metastasizes to distant organs. Remarkably, epigenetic alterations, including DNA methylation, histone modifications, and alterations in non-coding RNAs, can profoundly impact gene function in PC [90]. Elevated expression of *GAS41* has been observed in clinical PC specimens and mouse models, and the expression level of *GAS41* is associated with PC cell growth, migration, and invasion [41]. Mechanistic investigations have revealed that GAS41 interacts with β-catenin and acts as a positive regulator to activate β-catenin/TCF signaling to promote PC cell growth and metastasis [41, 91, 92]. It is important to note that GAS41 has previously been shown to promote H2A.Z deposition via recognition of histone acetylation [31]. Recent studies have reported that *H2A.Z.2* is overexpressed in human PC tissues and cell lines, and exogenous expression of *GAS41* or *H2A.Z.2* promotes NOTCH and NOTCH-mediated cancer cell stemness and GEM resistance [42]. However, a number of questions remain to be addressed. For example, what is the mechanism by which GAS41 promotes the deposition of ac H2A.Z.2. In summary, the association of GAS41 with PC is mediated through the β-catenin/TCF pathway and the NOTCH pathway (Fig. 3). Understanding the intricate mechanisms involving GAS41 in PC progression provides valuable insights for developing targeted therapeutic strategies against this devastating disease.

### GAS41 in gastric cancer (GC)

Gastric cancer (GC) is a significant global concern, with the majority of cases being diagnosed at stage IV of the disease, and poor prognosis [93]. Late-stage diagnosis and high mortality rates highlight the urgent need for novel therapeutic targets in GC. Claudin-18.2 [94], inhibitors of the fibroblast growth factor receptor 2 (FGF2) pathway [95], and combinations of anti-angiogenesis with immune checkpoint blockade are three recognized therapeutic targets for GC [96]. It has been established that *GAS41* is highly expressed in GC tissues and cell lines, and its increased expression has been linked to enhanced cell proliferation and attenuated apoptosis through activation of the Wnt/β-catenin signaling pathway [43, 70]. Furthermore, an analysis of five GC cell lines and 135 GC primary tumor samples revealed that patients with GAS41 overexpressing tumors have lower overall survival rates, and cell lines with GAS41 knockouts exhibit significantly increased chemosensitivity to CDDP (Cisplatin) and L-OHP [44]. In summary, GAS41 regulates the survival of GC cells by activating the Wnt/β-catenin signaling pathway (Fig. 3). The role of *GAS41* as a prognostic factor and potential therapeutic target highlights its contribution to tumor malignancy in GC*.* Therefore, modulating *GAS41* expression holds promise as an effective therapeutic approach for GC.

### GAS41 in colorectal cancer (CRC)

Colorectal cancer (CRC) ranks as the second most common cancer in women and the third most common cancer in men [97]. Globally, approximately 10% of cancer cases and cancer-related deaths can be attributed to CRC [98]. The majority of CRC cases arise from stem cells or stem cell-like cells [99], resulting from the accumulation of genetic and epigenetic alterations. An analysis of *GAS41* expression in 85 pairs of CRC and paracancerous tissues reveals that inhibition of GAS41 expression leads to cell cycle arrest in the G0/G1 phase and a significant increase in apoptotic cell numbers [45]. Additionally, a recent discovery unveiled that miR-218 inhibits cytoprotective autophagy by targeting GAS41, thereby sensitizing CRC cells to apoptosis induced by Oxaliplatin (L-OHP) [46]. MiRNAs have been extensively implicated in tumor proliferation, invasion, angiogenesis, and drug resistance [100], and dysregulation of several miRNAs has been reported in CRC [101, 102]. These findings represent a potential breakthrough in the diagnosis and treatment of colon cancer. Collectively, these studies indicate that GAS41 may serve as a critical modulator of proliferation and apoptosis in CRC cells and could regulate drug sensitivity in CRC through miRNAs (Fig. 3).

### GAS41 in uterine leiomyomas (ULs)

Uterine leiomyomas (ULs) represent the most prevalent benign gynecological tumors observed in women of reproductive age and postmenopausal women [103]. Abnormalities in various epigenomes have been identified in ULs, suggesting their involvement in the development and growth of these tumors [104]. In a recent clinical study utilizing genome-wide datasets to analyze UL origins, somatic mutations in six genes encoding the SRCAP histone loading complex were identified as biomarkers [50]. Notably, germline mutations in *GAS41* and *ZNHIT1* were found to predispose women to ULs. Tumors harboring these mutations exhibited impaired deposition of the histone variant H2A.Z [50]. Therefore, GAS41 may regulate the formation of uterine fibroids by influencing the deposition of the histone variant H2A.Z (Fig. 3). Additionally, a comprehensive evaluation of protein-coding genes in an extended exome sequencing cohort of 233,614 white European women further confirmed *GAS41* as a significant contributor to UL susceptibility [49]. However, the biological function of *GAS41* in ULs remains largely unknown, as current investigations are predominantly limited to bioinformatic analyses. Further research is needed to elucidate the precise role of GAS41 in ULs.

### GAS41 in liposarcoma (LPS)

Liposarcoma (LPS) is a rare malignant tumor characterized by adipocytic differentiation [105]. It is classified into four major subtypes: highly differentiated LPS (WDLPS, also known as atypical lipomatous tumors), dedifferentiated LPS (DDLPS), mucinous-like LPS (MLPS), and pleomorphic LPS (PLPS) [47]. Previous studies have suggested that *GAS41* may serve as a critical oncogene in atypical LPS [106]. Barretina et al. showed that the knockdown of GAS41 significantly reduced cell proliferation in DDLPS [107]. Moreover, recent literature has reported aberrant amplification of *GAS41* in atypical LPS [108]. Collectively, these findings suggest a potential active role for GAS41 in liposarcoma. However, the precise function of GAS41 in LPS and the underlying signaling pathways involved remain unclear.

### GAS41 in ovarian cancer (OC)

Ovarian cancer (OC) is a highly life-threatening malignancy affecting women worldwide, necessitating the urgent discovery of effective biomarkers [109]. This rapidly proliferating cancer exhibits temporary chemosensitivity, imposes pressure on internal organs, and exhibits a cure rate of only 30% [110]. An inherent challenge in OC treatment is that most patients are diagnosed with advanced-stage disease, and long-term chemotherapy often leads to drug resistance [111]. In a study by Kim et al., the analysis of drug resistance-associated transcription factors (TFs) in OC highlighted *GAS41* as a key transcription factor that induces chemoresistance through an intrinsic apoptosis-related pathway [48] (Fig. 3). To future elucidate the role and mechanism of GAS41 in the pathogenesis of OC, comprehensive investigations are warranted.

## Continued research on GAS41

**Fig. 4:**
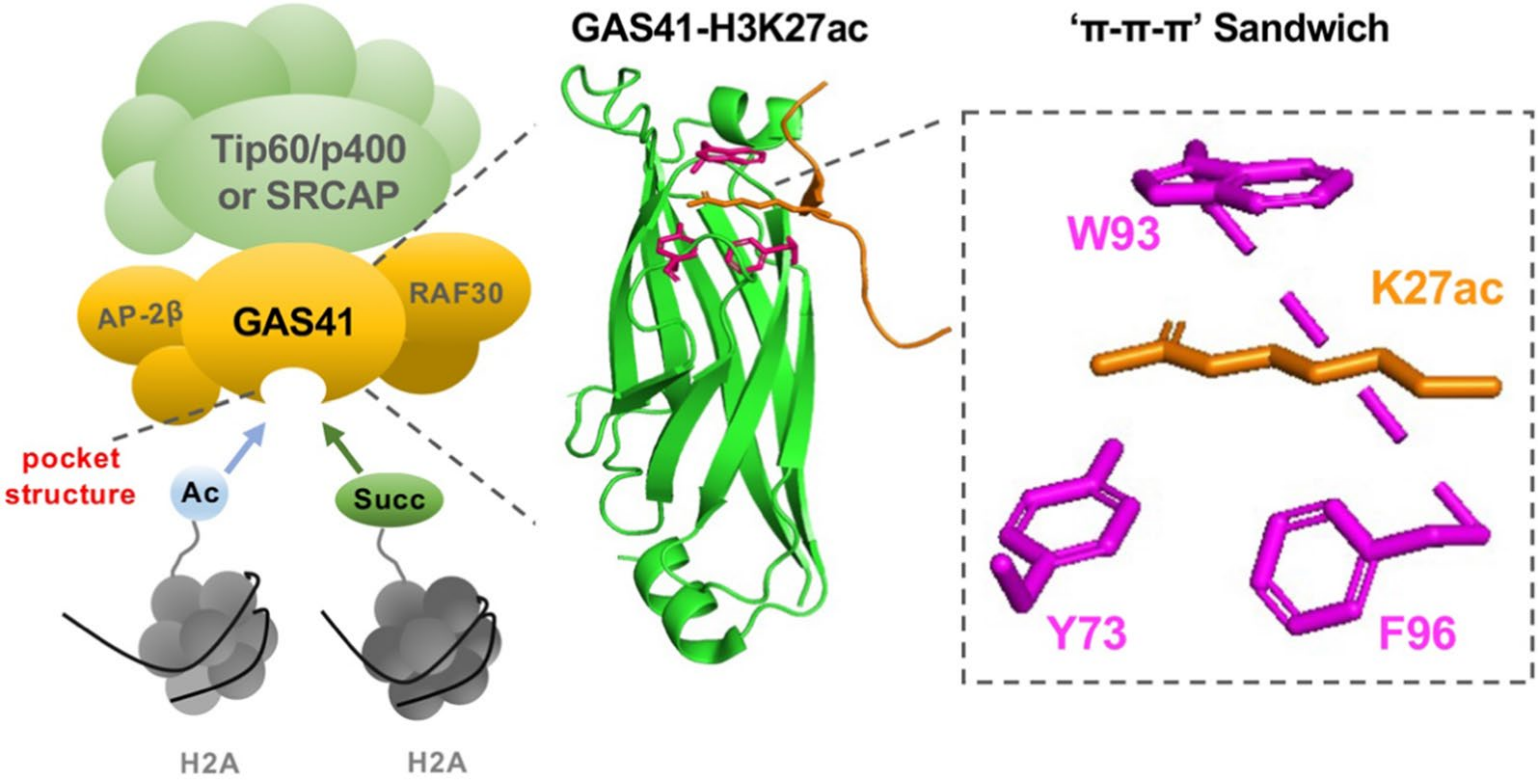
A working model for GAS41 in regulating carcinoma. GAS41-H3K27ac is derived from PDB (5xtz). Green indicates GAS41and orange is part of the H3 peptide. Pink represents the three key residues: W93, F96, and Y74

Cells employ a diverse repertoire of transcriptional regulatory proteins to finely modulate gene expression [32]. Studies have established the crucial role of recognizing post-translational modifications of histones in transcriptional regulation [112]. A relatively recent discovery, the YEATS domain proteins, constitute a family of epigenetic reader proteins [1, 2, 62, 113]. Dysregulation of epigenetic reader proteins is frequently observed in cancer, making them attractive targets for the development of small molecule inhibitors [27, 114–116]. Listunov et al. have developed a nanomolar active dimeric analog that disrupts the interaction between GAS41 and acetylated histone H3 [27]. Subsequently, Londregan et al. have identified selective small molecule inhibitors with a bias toward the YEATS domain [117]. Biochemical investigations have shown that the YEATS domain of GAS41 recognizes histone acetylation, benzoylation, succinylation, and crotonylation [31, 62–64, 118] (Table 1). Notably, Liu et al. assume a three-phase traffic-light system model describing three different H3K27 modifications as distinct chromatin states for gene transcription [63]. H3K27me3 (stop) marks for transcriptional silencing, H3K27cr (pause) for transcriptional repression, and H3K27ac for transcriptional activation (go) [63]. Aberrant patterns of histone acylation are closely linked to human cancers [119], influencing gene expression and cell signaling processes within tumors [7, 120]. Understanding the mechanisms underlying histone acylation recognition and deposition is vital for developing effective anti-cancer strategies [7]. While the functional aspects of acetylation are relatively well-established and comprehensive, studying the functional disparities between non-acetylated and acetylated histones remains challenging. Histone Kcr levels are reduced in prostate cancer, HCC, GC, and kidney cancer [121, 122], while they are increased in intestinal cancer, thyroid cancer, esophagus cancer, PC, and NSCLC [122, 123]. Thus, the level of histone Kcr directly or indirectly affects the characteristics of cancer cells. Histone Ksucc is implicated in PC [124], esophageal squamous cell carcinoma [125], GC [126], renal cell carcinoma [127], HCC [128], CRC [129], and glial blastoma [130]; however, its role in tumor development is context-dependent. All the evidence suggests that GAS41 holds significant potential for association with various cancers through the recognition of acylated modifications (Fig. 4, Additional file 2: Fig. S2).

**Table 1:** Summary of recognizable histone modifications of GAS41

Acylation	Histone lysine	Binding affinity KD(μM)	Target gene	Specificity	Association with cancer	References
Kac	H3K23acK27ac	13.6 ± 0.3	Unknow	Diacetylation	Unknow	[63]
	H3K27ac, H3K14ac	32.7, 13	*H2AFV*,* H2AFZ*	H2A.Z deposition	NSCLC	[31]
Kbz	H3K27bz	62.96 ± 11.24	Unknow	Open-end reader pocket	Unknow	[65]
Ksucc	H3K122succ	2.937 ± 0.21 (pH6.0), 48.34 ± 3.8 (pH7.0)	*p21*	pH dependent	pH imbalance	[67]
Kcr	H3K27cr	22.9	*p21*	Three-phase traffic light system	CRC	[57]

## Conclusions and outlooks

**Fig. 5:**
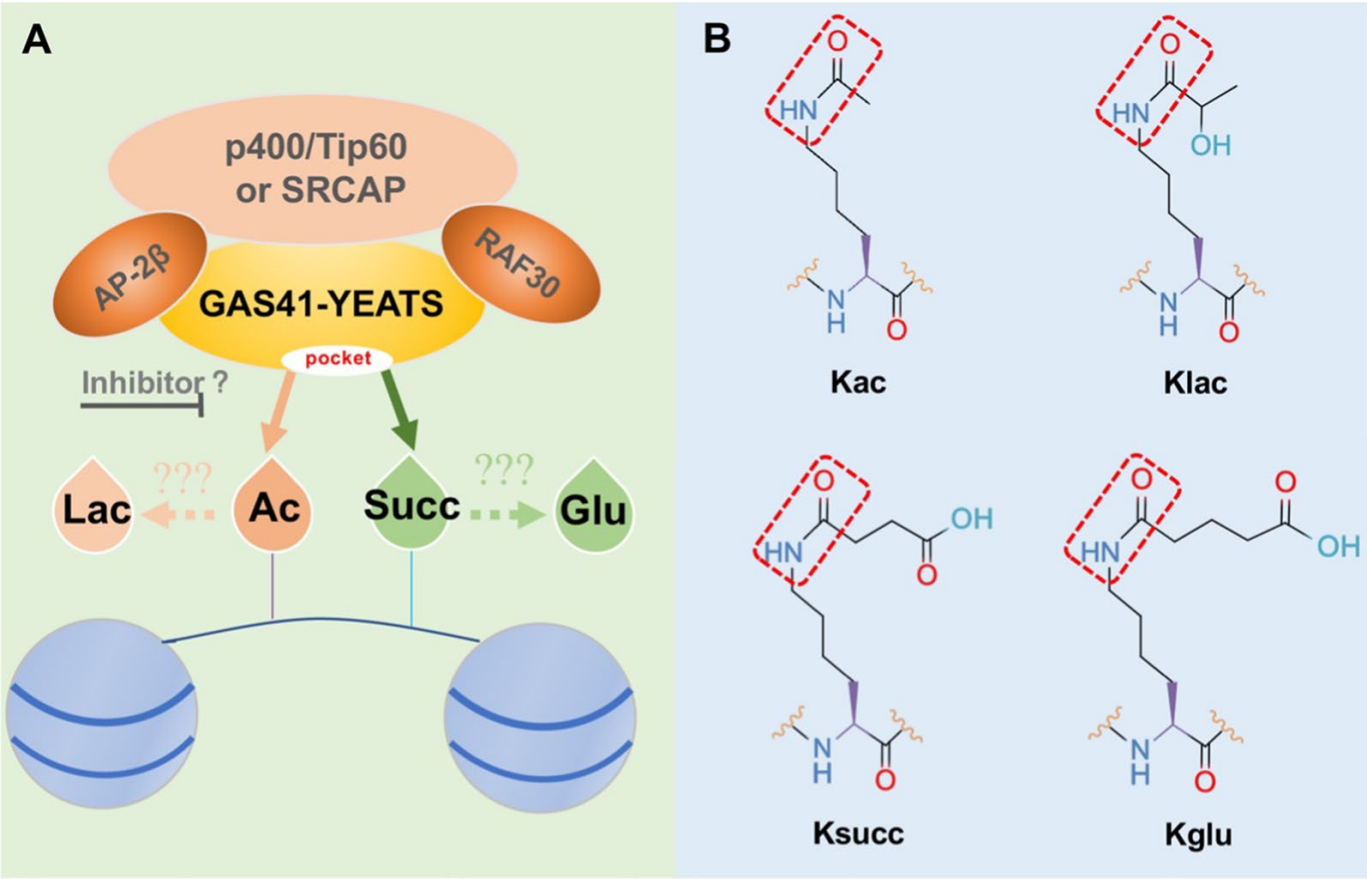
GAS41 plays a role in various cancers by reading novel modifications. Schematic representation of the hypothesis that GAS41 recognizes novel histone acylation modifications. **A** GAS41 recognizes the histone modifications through the YEATS domain. **B** Chemical structural formulae of some lysine acylation modifications. *Lac* lactylation modification; *Glu* glutarylation modification

Cancer cells rely on chromatin regulatory pathways and transcriptional mechanisms to maintain an oncogenic state, making these processes attractive targets for drug development. Metabolic remodeling is a hallmark of cancer cells, leading to abnormal accumulation of metabolites. Covalent modification of proteins through lysine acylation by various metabolites contributes to epigenetic remodeling. Aberrant epigenetic landscapes in cancer cells often exploit chromatin mechanisms to activate oncogenic gene expression programs. The recognition of histone modifications by "reader" proteins is a key process in these events. As a representative of the reader module for short-chain lysine acylation, the YEATS domain plays a critical role in lysine acylation biology, serving as a link between metabolism and gene regulation. GAS41, a novel epigenetic reader of acylated modifications, holds significant potential for association with pathophysiological processes in relevant cancers through its recognition of acylated modifications (Additional file 2: Fig. S2). Histone lactylation (Klac) is a recently discovered component of the human cellular epigenetic landscape (Fig. 5B), sensitive to both exogenous and endogenous lactate levels [131]. Elevated lactate levels in the tumor microenvironment (TME) lead to increased intracellular lactylation, and both lactylation and lactate have been considered for cancer therapy [132]. Lactylation has been implicated in tumor immune escape mediated by tumor-infiltrating myeloid cells (TIMs) [133]. Lactate enhances the stemness of CD^8+^ T cells and improves anti-tumor capacity [134]. Controlling the glycolytic switch marked by lactylation presents therapeutic opportunities for cancer [135]. Lysine glutarylation (Kglu), another newly characterized protein lysine modification (Fig. 5C), exhibits diverse functions in eukaryotic cells [136–139]. However, the role of Kglu as a reader in cells and its contribution to cancer remains unclear. Given its chemical structural properties resembling Kac and Ksucc modifications, GAS41 is likely to act as a reader for Klac and Kglu (Fig. 5). Currently, there are limited studies on GAS41’s recognition of acylated modifications in cancer cells. Therefore, establishing a comprehensive framework to understand the complexity and specificity of GAS41 is crucial. Moving forward, investigating GAS41’s recognition of histone acylation modifications to target downstream oncogenes and developing small molecule inhibitors to disrupt this process will be promising areas of research. In summary, further in-depth exploration is required to enhance our understanding of GAS41 as a signaling transduction protein and transcription factor.

### Supplementary Information

**Additional file 1: Figure S1**. An overview of the *GAS41* involved in cancer.

**Additional file 2: Figure S2**. A working model for GAS41.

## Data Availability

Not applicable.

## Abbreviations

ADA3: Alternation/deficiency in activation-3
AF9: ALL1-fused gene from chromosome 9
AP-2β: Activating enhancer-binding protein 2-beta
ATAC: Ada-two-A-containing
BAF: BRG1-associated factor
BC: Breast cancer
CRC: Colorectal cancer
CDDP: Cisplatin
DDX3: DEAD-box protein 3
DDLPS: Dedifferentiated LPS
DOT1L: Histone-lysine N-methyltransferase
EMT: Epithelial-mesenchymal transition
ENL: Eleven-nineteen-leukemia
FGF2: Fibroblast growth receptor 2
GAS41: Glioma amplified sequence 41
GC: Gastric cancer
GCN5: General control non-repressed protein 5
GEM: Gemcitabine
HCC: Hepatocellular carcinoma
Kac: Lysine acetylation
Kbz: Benzoylation
Kcr: Lysine crotonylation Klac Lysine lactylation
Kglu: Lysine glutarylation
Ksucc: Lysine succinylation
L-OHP: Oxaliplatin
LPS: Liposarcoma
Me: Methylation
MLL: Mixed lineage leukemia
MLLT1: Mixed-lineage leukemia translocated to 1
MLLT3: Mixed-lineage leukemia translocated to 3
MLPS: Mucinous-like LPS
MOZ: Monocytic leukemia zinc
NSCLC: Non-small cell lung cancer
OC: Ovarian cancer
PAF1: Polymerase-associated factor 1
PC: Pancreatic cancer
PLPS: Pleomorphic LPS
PP2Cβ: Protein phosphatase 2C protein phosphatase 2C β
p400: E1A-binding protein p400
RAP30: RNA polymerase-associated proteins 30
SEC: Super elongation complex
SRCAP: SNF2-related CBP activator protein
TACC: Transforming acidic coiled-coil 1
TAK1: Transforming growth factor beta-activated kinase 1
TCEA1: Transcription elongation factor A protein 1
TFs: Transcription factors
TIP60: Tat-interactive protein 60
TME: Tumor microenvironment
TRRAP: Transcription/transformation domain-associated protein
ULs: Uterine leiomyomas
WDLPS: Highly differentiated LPS
ZEB1: Zinc finger E-box-binding homeobox 1
ZZZ3: Zinc finger ZZ-type containing 3
