# Supplementary figures and images for: Unveiling the role of GAS41 in cancer progression
Source: Cancer Cell Int. 2023 Oct 18;23:245. doi: 10.1186/s12935-023-03098-z (PMC10583379; doi:10.1186/s12935-023-03098-z)

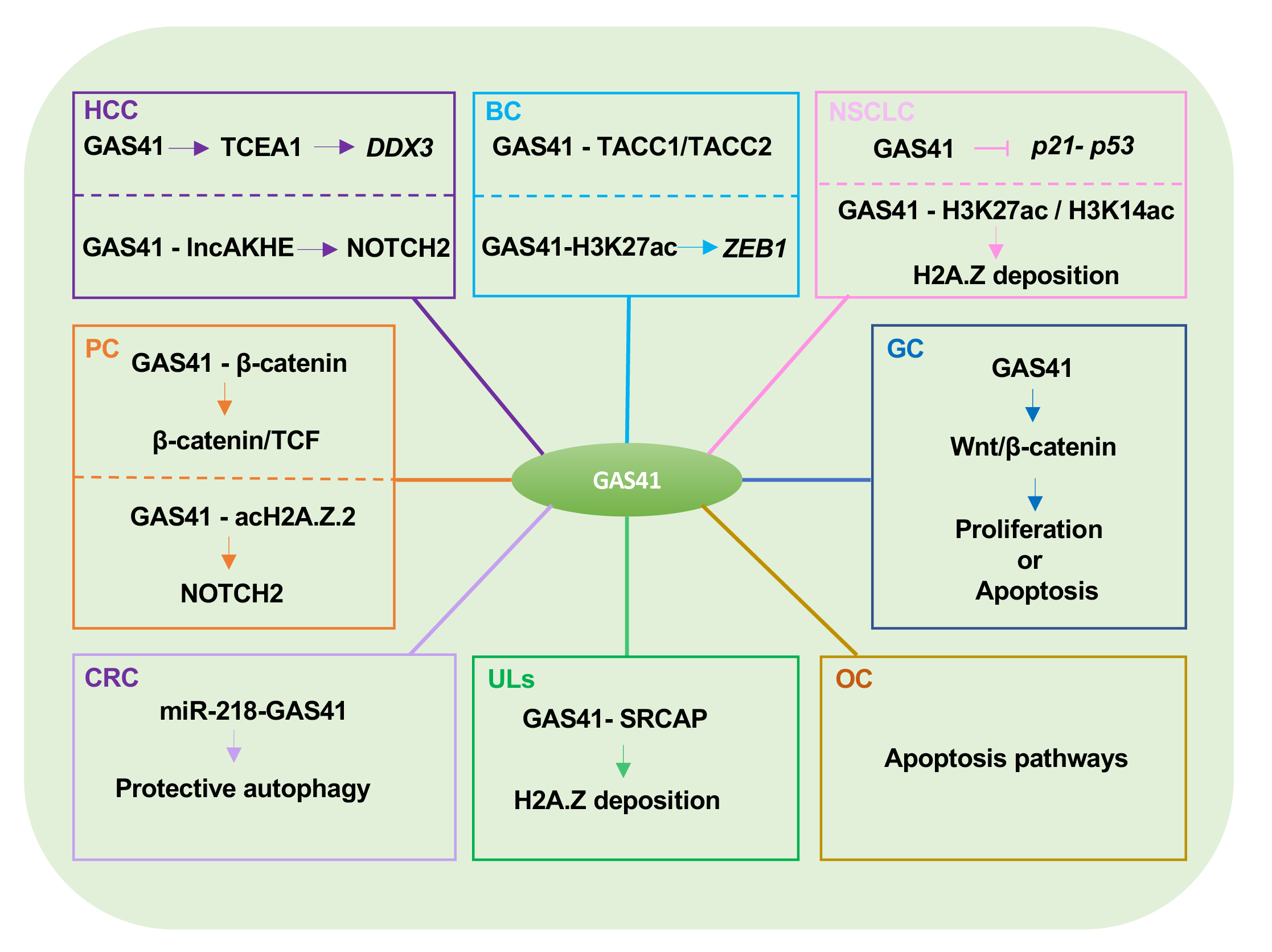

Supplement: Supplementary file 1 — Additional file 1: Figure S1. An overview of the GAS41 involved in cancer. [file 12935_2023_3098_MOESM1_ESM.jpg]

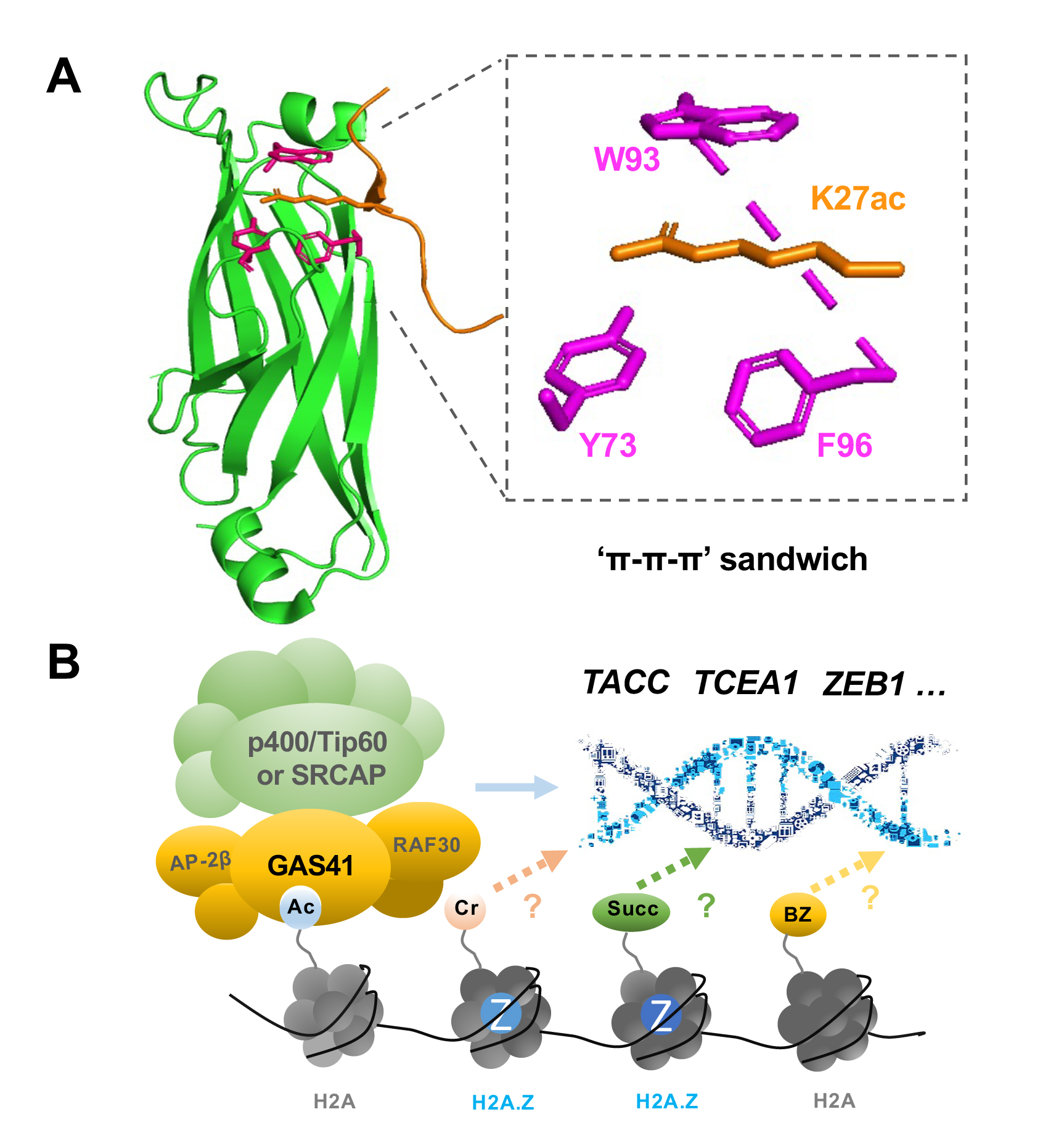

Supplement: Supplementary file 2 — Additional file 2: Figure S2. A working model for GAS41. [file 12935_2023_3098_MOESM2_ESM.jpg]
